# Preparation for Childbirth: Coping with the Fear of Childbirth

**DOI:** 10.3390/healthcare11040480

**Published:** 2023-02-07

**Authors:** Juan Carlos Sánchez-García, Jonathan Cortés-Martín, Raquel Rodríguez-Blanque

**Affiliations:** 1 Andalusia Research Plan, Junta de Andalucía, Research Group CTS1068, School of Nursing, Faculty of Health Sciences, University of Granada, 18071 Granada, Spain; 2 School of Nursing, Faculty of Health Sciences, University of Granada, 18071 Granada, Spain; 3 Hospital Universitario Clínico San Cecilio, 18016 Granada, Spain

Pregnancy is a vital event in a woman’s life that involves not only important physical changes, but also psychological changes. There are feelings of ambivalence towards the new state, fear during pregnancy and when coping with childbirth, and later, difficulty in accepting the maternal role [1].

Adaptation to all these changes generates feelings of stress and anxiety that increase as the pregnancy progresses, as reported by 80% of pregnant women [2].

However, for some women, childbirth can pose a threat, causing harm and pain that can generate intense fear. In addition, it should be noted that this feeling of fear usually generates a maternal psychological discomfort, which causes an increase in catecholamines, preventing the normal secretion of oxytocin that is meant to trigger the correct evolution of labor, and thus, leading to longer labor; therefore, fear is considered a risk factor for various complications during childbirth [3,4]. According to the literature, 53% of women have an excessive fear of childbirth, with the main causes identified as the fear that their child will suffer an injury during labor or die, or be born with a malformation [5].

The reality is that clinical attention during pregnancy and childbirth has improved ostensibly over the years, leading to the early detection of anomalies in fetuses, and greater safety when performing surgery during childbirth. However, there is no evidence of a decrease in women’s fear when facing childbirth, a fact that makes us think that despite scientific advances in the way of approaching childbirth, there are other factors that influence women’s feelings during this period.

Pain is not in itself a pathological factor, but a physiological condition caused by the contraction of the smooth muscles of the uterus to guide the fetus out [6]. When pain is not adequately treated, it can adversely affect maternal and fetal health, with adverse effects appearing in the respiratory, cardiovascular, neuroendocrine, and limbic systems [7,8]. Therefore, pain management can control the occurrence of these adverse effects, along with providing symptomatic pain relief.

Fear of pain and childbirth is multifactorial; however, the risk factors that have been most associated with its development include the following: early gestation in older women (>40 years), low socioeconomic status, smoking, anxiety and/or depression prior to or during pregnancy, high-risk gestation, lack of support, pregnant women with a history of sexual abuse, and nulliparity or previous cesarean section [9,10].

The consequences of the fear of childbirth can generate insecurity, frustration, and a lack of self-efficacy in the woman’s ability to give birth [11]. Likewise, a previous negative birth experience generates a greater risk of fear in subsequent pregnancies [12] and increases the chance of requesting an elective cesarean section (without medical indication) [13]. Similarly, it may increase the risk of emergency cesarean section [14], complications during labor and/or expulsive dystocia [15], and the duration of labor [8].

Although labor pain is an experience that is part of the natural process and occurs for a limited time, it is perceived and experienced differently by each woman [16]. It is described as one of the most intense feelings of pain that a woman can experience in her lifetime [17] and is subjective.

Many techniques and coping strategies have been identified to reduce and control the pain and fear that a woman may experience during a painful experience; among them, we find as the main method of pain relief and control in Western countries the use of epidural analgesia, but we can find other alternatives, such as the use of hydrotherapy, massage, acupuncture techniques, relaxation, breathing, etc., which together with prenatal education that addresses the psychological changes and fear of childbirth can liberate the woman emotionally, making her feel more confident, calm, and more able to control her own childbirth.

When a woman can cope with the pain of childbirth effectively, she begins motherhood with a positive experience, experiences the happiness and satisfaction of actively participating in the birth of her baby, and engages in practices together with health professionals during the postpartum period.

While always respecting each woman’s expression of pain, it is necessary to personalize the resources available for pain relief and management during childbirth. For this, the first step is to be informed of all resources, both pharmacological and non-pharmacological; to know their efficacy; and to make them available to the woman so that she becomes involved in her childbirth process.

The use of non-pharmacological analgesic methods, which are safer for both the mother and the fetus, can be an alternative to pharmacological analgesic methods that can carry various risks, as is the case with epidural analgesia [18,19,20,21].

Through this topic, we try to address various methods and techniques that facilitate the relief and control of pain and fear during childbirth, facilitating the empowerment and active participation of women in their pregnancy, childbirth, and puerperium process.
